# Microstructural Evolution of Cold-Rolled AA7075 Sheet during Solution Treatment

**DOI:** 10.3390/ma13122734

**Published:** 2020-06-17

**Authors:** Lu Wang, Xiaofang Yang, Joseph D. Robson, Robert E. Sanders, Qing Liu

**Affiliations:** 1 International Joint Laboratory for Light Alloys (Ministry of Education), College of Materials Science and Engineering, Chongqing University, Chongqing 400044, China; wanglu@cqu.edu.cn (L.W.); qingliu@cqu.edu.cn (Q.L.); 2 School of Materials, The University of Manchester, Manchester M13 9PL, UK; joseph.robson@manchester.ac.uk; 3 Shenyang National Laboratory for Materials Science, Chongqing University, Chongqing 400044, China; 4 Key Laboratory for Light-weight Materials, Nanjing Tech University, Nanjing 210009, China

**Keywords:** Al-Zn-Mg-Cu alloy, heating rates, dissolution, recrystallization, pinning effect

## Abstract

The influence of heating rate on the microstructural evolution of a cold-rolled AA7075 alloy sheet during solution heat treatment was examined using electrical conductivity, scanning electron microscopy, X-ray diffraction, transmission electron microscopy and electron backscatter diffraction. The results indicate that the dissolution of soluble phases takes place during the heating process. The heating rates affect the dissolution process of soluble phases, and these phases completely dissolve into matrix after solution treatment. Recrystallized and elongated grains are produced after solution treatment by both fast and slow heating rates, while the grains of the rapidly heated sample are much finer. The elongated grains are attributed to the difference in the pinning pressure of boundary migration between the rolling and normal directions. The {111}<110> texture, as well as typical recrystallization textures, were found in both fast and slowly heated samples after solution treatment, but the textures, especially the {111}<110> component in the slow-heated sample, are much stronger, leading to an anisotropy in the tensile properties after artificial aging.

## 1. Introduction

Al-Zn-Mg-(Cu) alloys have been widely used in aircrafts due to their high static strength and fracture toughness [1,2,3]. Recently the need to produce lightweight structures in automobiles has driven extensive efforts to apply them in automotive body components [4,5,6,7]. One of the oldest 7xxx alloys, AA7075 alloy, introduced by Alcoa in 1943, has been evaluated as an alternative for strength-critical auto parts. The use of 7xxx alloys will likely require the modification of numerous automotive forming [7,8,9], joining, or finishing processes. For example, hot, rather than cold, stamping might be employed, or different bake-hardening cycles could be necessary [10,11]. The continuous solution treatment of coils (rapid heating) is often applied during auto industrial processing to guarantee production efficiency [12], while the batch processing of individual blanks (slow heating) is often done in the aviation industry. The effects of heating rate during solution treatment on microstructures and textures have been studied in Al-Cu-Mg [13] and Al-Mg-Si-Cu alloys [14]. However, few studies have been conducted on Al-Zn-Mg-(Cu) alloys.

Before solution treatment Al-Zn-Mg-(Cu) alloys contain constituents, dispersoids and equilibrium η precipitates. The constituents and dispersoids are insoluble, while the precipitates dissolve during solution treatment. The constituents known as Fe-bearing particles, such as Al_7_Cu_2_Fe, form during solidification and have the largest dimension in Al-Zn-Mg-(Cu) alloys. The constituents are nondeformable during hot and cold working and large strain gradients are produced around these particles. Consequently, the constituents can act as recrystallization nucleation sites due to a particle-stimulated nucleation (PSN) mechanism [15,16]. The dispersoids in Al-Zn-Mg-(Cu) alloys are sub-micron compounds formed from the additions of Cr, Mn or Zr during homogenization. The number and size of the dispersoids are determined by the chemical composition, thermal history and severity of deformation. Dispersoid particles are distributed heterogeneously after deformation and are present in the form of alternating bands or layers. The growth of recrystallized grains is obstructed by these bands or layers and elongated grains are commonly produced in Al-Zn-Mg-(Cu) alloys [17]. The precipitates in Al-Zn-Mg-(Cu) alloys after hot working are equilibrium η phases and their size depends on the prior thermal process and hot or cold working history. Equilibrium η phases may grow up to micron scale under favorable conditions; however, equilibrium η phases are soluble and can completely dissolve during solution treatment. Both the dispersoids and precipitates have been reported to interact with dislocations [18], as well as sub-grain and grain boundaries [18,19,20,21,22] when recovery and recrystallization occur. The coexistence of constituents, dispersoids and precipitates makes the recovery and recrystallization behavior highly complex during solution treatment.

In the present paper, the initial microstructures in a cold-rolled AA7075 sheet were examined using scanning electron microscopy (SEM) and transmission electron microscopy (TEM). The effects of heating rates on the microstructures and textures were studied using electrical conductivity (EC), SEM, electron backscatter diffraction (EBSD) and X-ray diffraction (XRD). The dissolution of the equilibrium precipitates, recovery, and recrystallization during the heating process of the solution treatment are discussed in detail.

## 2. Materials and Methods

The chemical composition of the AA7075 alloy sheet is listed in Table 1. Small direct chilled (DC) ingots were cast, homogenized at 450 °C for 24 h and hot rolled to a thickness of 10 mm from 40 mm. The plates were heated to 420 °C and soaked for 15 min before hot rolling. Air cooling was carried out after hot rolling. Small strips were subsequently cold rolled to a final thickness of 3 mm with a reduction of 70%.

**Figure 1:**
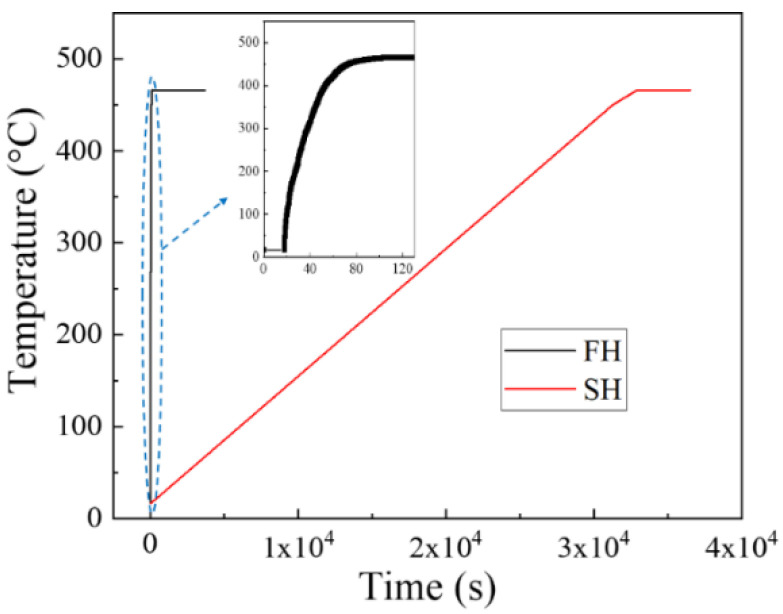
The measured heating curves for both FH and SH samples.

Specimens cut from the cold-rolled sheet were solution treated at 475 °C for 1 h in a circulating air furnace. Two heating rates were used during the heat-up: Fast heating (FH) was performed by putting samples in the furnace, pre-heated to the solution temperature (475 °C). To make the slow heating (SH) samples, samples were placed in the furnace at room temperature and heated at 50 °C/h to 475 °C. The heating rates for both the FH and SH samples were measured using a thermocouple. The heating curves are shown in Figure 1 and the inset figure is the rapid heating curve in the very early stage. The measured heating rate for the FH sample is approximately 325 °C/min and the heating rate for the SH sample is as expected (50 °C/h).

Interrupted heating was carried out to study the dissolution of soluble phases during the heating ramp. For the fast heating rate, in particular, interrupted samples were obtained by counting the soaking time. To ensure the accuracy of the soaking time, the samples, quenched at different temperatures, were prepared one by one. For example, for 20 s at 475 °C (air furnace temperature), the 3 mm samples reached a temperature of 300 °C, according to the measured heating curve. Thus, the sample quenched at 300 °C was obtained by soaking for 20 s in the pre-heated furnace (475 °C), followed by the immediate quenching into water without any delay. The samples at other temperatures were then prepared one by one by repeating these procedures after the furnace temperature reached 475 °C again. The ECs of all samples were measured within 5 min after quenching using a Fischer Sigmascope SMP10 electrical conductivity tester (Fischer Technology Inc., Windsor, CT, USA) after calibration with a standard sample. All of the samples were stored in a freezer (−80 °C) to prevent natural aging after the EC measurement.

**Figure 2:**
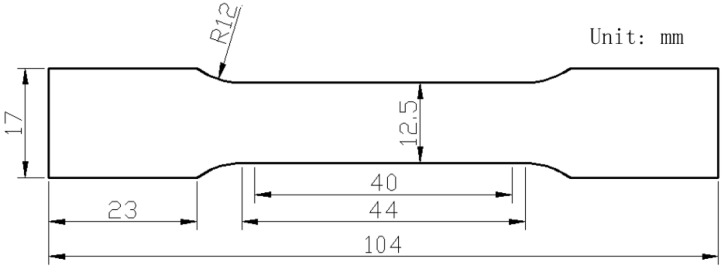
The dimensions of tensile samples.

The fully solutionized samples were artificially aged for 24 h at 120 °C (T6) in an oil bath. The tensile samples were cut from the aged sheet along the rolling direction (RD), 45° from the RD and the transverse direction (TD). The dimensions of the tensile samples are shown in Figure 2. The tensile samples were then tested on an Instron static testing machine at a strain rate of 0.005 s^−1^. Three tests were performed for each condition and the average strengths were calculated.

To characterize the second phase particles of the cold-rolled sheet and the heat-treated samples, SEM observations were conducted on the rolling direction (RD)–normal direction (ND) plane of samples after careful grinding and mechanical polishing. The samples were then prepared for EBSD by electropolishing at −15 °C and 20 V with a solution of 10% perchloric acid and 90% ethanol. Both SEM and EBSD were performed on a Tescan Mira 3 field emission gun (FEG)-SEM (Tescan Corporation, Brno, Czech Republic). The EBSD data were processed using HKL Channel 5 software (Version 5.0.9.0, Oxford Instruments, Abingdon, UK) to determine the grain size and recrystallized fraction. The texture components were measured on the rolling plane at mid-thickness using a Bruker X-ray diffractometer (Bruker Corporation, Billerica, MA, USA) with a cobalt radiation source and the raw data were analyzed using Atex software (Version 1.39, University de Lorraine, Metz, France) [23]. The TEM samples were carefully ground to ~80 μm and then punched out. The twin-jet electropolishing was performed on a Struers TenuPol-5 machine (Struers Inc., Copenhagen, Denmark) at −30 °C and 15 V with a solution of 30% nitric acid and 70% methanol.

Dislocation density was obtained from X-ray line profile measurements [24]. The X-ray diffraction profiles were taken on a Proto AXRD (Stanley Black & Decker Inc., New Britain, CT, USA), equipped with a Cu radiation source (λ = 0.154 nm). The operation was carried out at 30 kV and 20 mA in a step-scan mode, and the 2θ was scanned in the range of 30°–145° with a step size of 0.02° and a dwell time of 8 s. The surface layer of each sample was removed by electropolishing to avoid machining effects during the sample preparation. The raw profiles were fitted using the convolutional multiple whole profile (CMWP) fitting method. The instrumental profiles of LaB_6_ for CMWP fitting were measured on the same machine with the same setup. The crystallographic parameters of aluminum, such as lattice constant (0.405 nm) and the absolute value of the Burgers vector (0.286 nm) were inputted for CMWP fitting. The average dislocation contrast factor for fitting was set as 0.2. The peaks from the second phase particles (Al_7_Cu_2_Fe and η) were also considered. Details about the CMWP fitting technique can be found in [25,26,27].

## 3. Results

### 3.1. Microstructures in the Cold-Rolled Sheet

**Figure 3:**
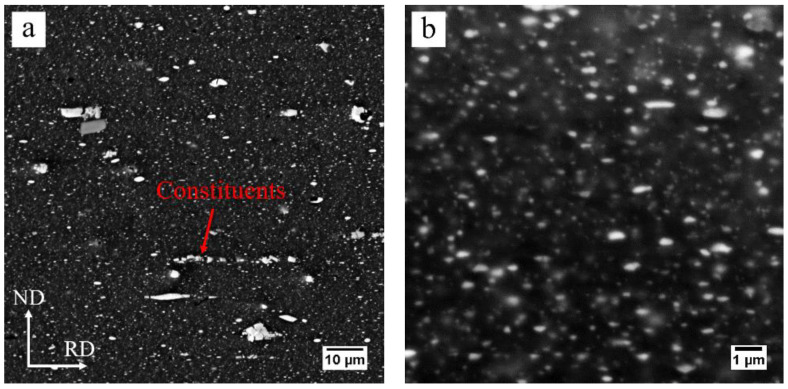
SEM images of the cold-rolled AA7075 sheet: (**a**) low magnification, (**b**) high magnification.

The EC of the cold-rolled sample was measured as 45.4% International Annealed Copper Standard (IACS), compared with the ~32.5% IACS for the solution-treated 7075 alloy, indicating that a high fraction of the solute is present in the form of second phase particles. These particles were observed using SEM, as shown in Figure 3. The low magnification SEM image in Figure 3a clearly shows that the constituents, normally identified as Al_7_Cu_2_Fe in 7075 alloy, were fragmented and aligned with the direction of cold rolling. The high magnification SEM image, as shown in Figure 3b, shows that a large number of smaller particles, known as dispersoids and equilibrium precipitates (η phases) in 7075 alloy, were observed.

**Figure 4:**
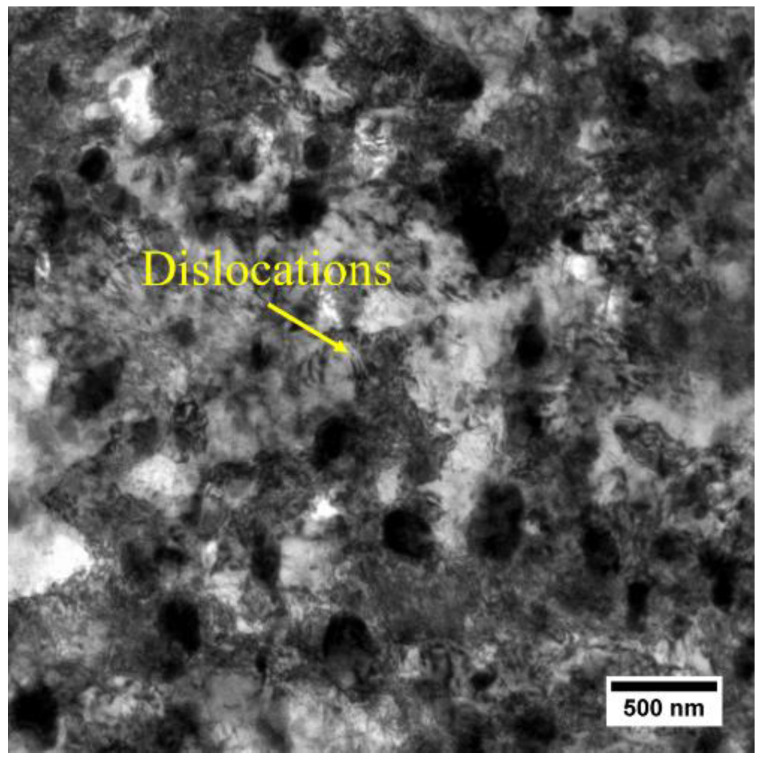
TEM-BF image of the cold-rolled sample.

**Figure 5:**
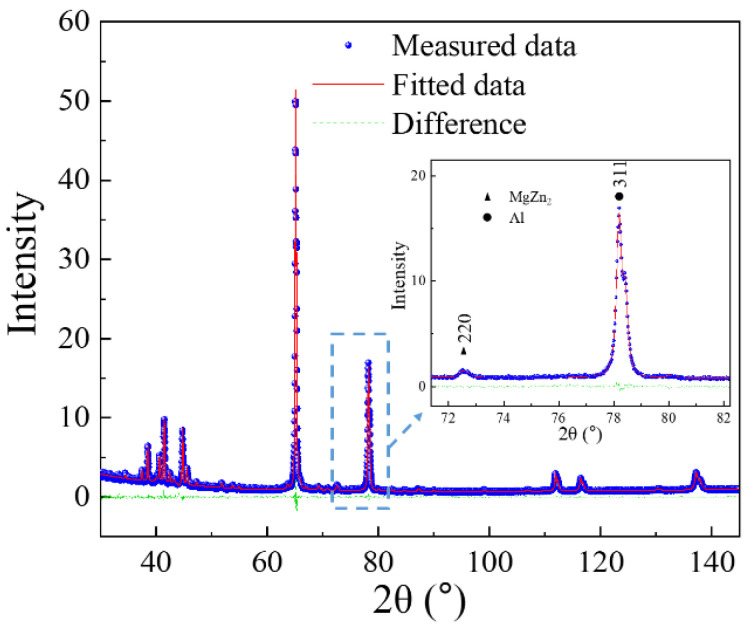
The diffraction pattern and the fitted pattern of the cold-rolled sample.

The high density of dislocations after cold rolling is observed in the TEM bright field (BF) image, shown in Figure 4. The dislocation density is difficult to determine from the TEM-BF image as the dislocations tangle up with each other and small particles. However, the fitting of X-ray line profile data can be used for obtaining the dislocation density [28]. The diffraction patterns, including the measured patterns, the fitted patterns and the differences, are shown in Figure 5. The zoomed figure in Figure 5 shows that the peaks from second phases (η) were also fitted in the CMWP procedure. The fitting results show that the dislocation density of the cold-rolled AA7075 sheet is 3.28 ± 0.43 × 10^14^ m^−2^, providing the driving force (stored energy) for recovery and recrystallization.

### 3.2. Dissolution of Equilibrium Precipitates (η)

**Figure 6:**
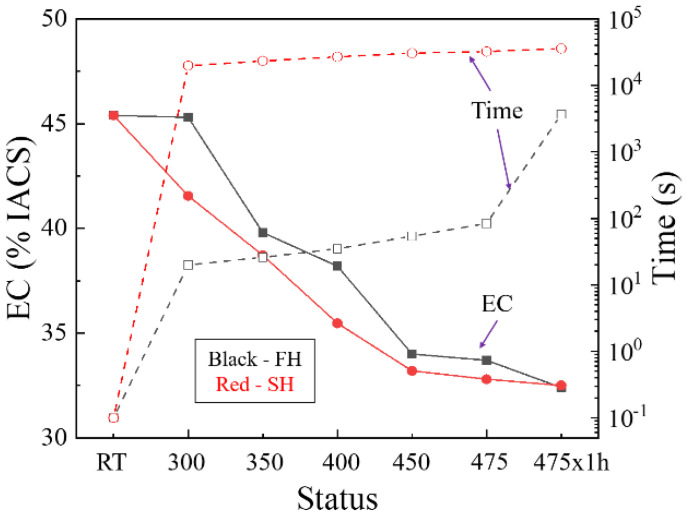
The EC evolution and the heating time corresponding to the temperature during the heat-up and solution treatment.

**Figure 7:**
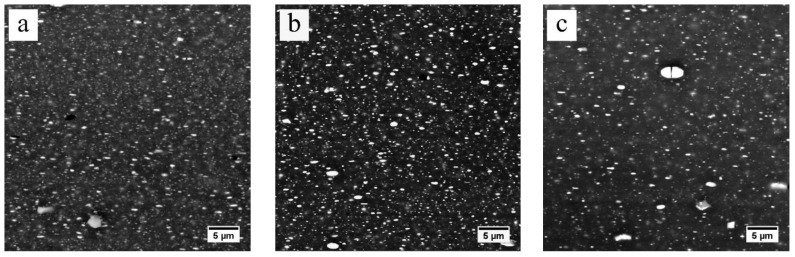

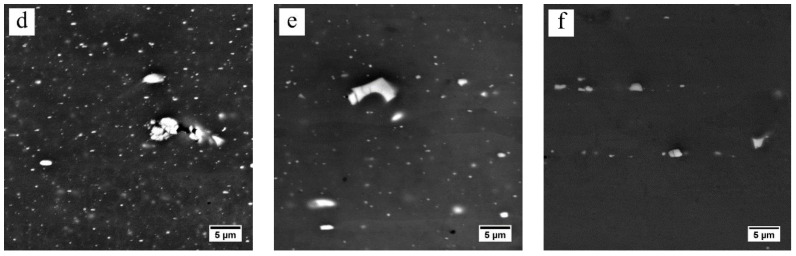
The SEM-BSE images of fast-heated samples quenched at (**a**) 300 °C, (**b**) 350 °C, (**c**) 400 °C, (**d**) 450 °C, (**e**) 475 °C and (**f**) 475 °C for 1 h.

**Figure 8:**
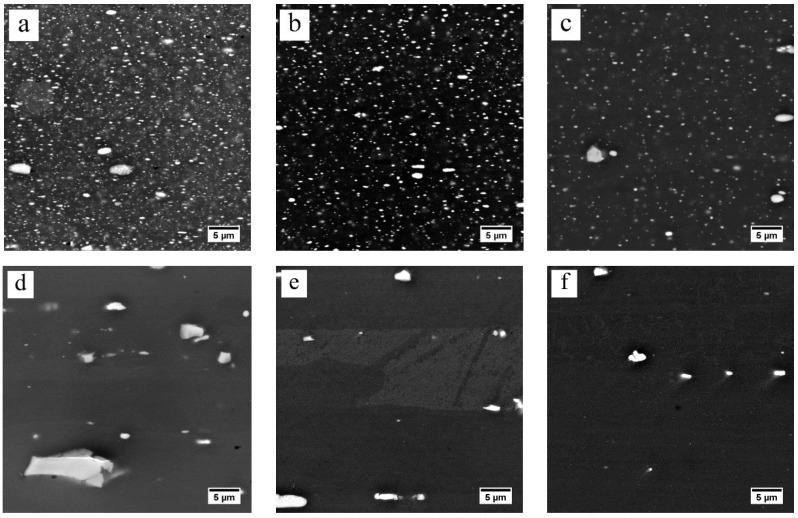
The SEM-BSE images of slow-heated samples quenched at (**a**) 300 °C, (**b**) 350 °C, (**c**) 400 °C, (**d**) 450 °C, (**e**) 475 °C and (**f**) 475 °C for 1 h.

Figure 6 shows the EC evolution and the heating time corresponding to the temperature during the heat-up and solution treatment. Since the constituents and dispersoids are insoluble, the change in EC (ΔEC) during solution treatment can be attributed to dissolution of the η phase. The dissolution of the η phase particles during fast and slow heating is also observed using SEM, as shown in Figure 7 and Figure 8, respectively.

During heating from room temperature to 300 °C, the slight decrease in EC for the FH samples indicates that little dissolution has occurred. The larger reduction in EC for the SH sample indicates that more η phase has dissolved, compared to the FH sample. However, the SEM observations of the FH sample, as shown in Figure 7a, and the SH sample, as shown in Figure 8a, quenched after heating to 300 °C, show that both the FH and SH samples have a large number of precipitates left. When the temperature goes up from 300 to 450 °C, the EC values of both the FH and SH samples in Figure 6 decrease rapidly. For example, the rapid heating reduces the EC value from 45.3% IACS at 300 °C to 34% IACS at 450 °C within ~2 min. The SEM-BSE images in Figure 7b–d and Figure 8b–d clearly show a rapid dissolution of the η particles during the fast and slow heating, which is consistent with the change in EC. Figure 7d shows that a few η phases were left after rapidly heating to 450 °C. However, very few η particles remained in the SH sample quenched at 450 °C, shown in Figure 8d, leading to a lower EC value. For both FH and SH samples, the EC decreased slowly in the following heat-up from 450 to 475 °C and the 1 h soak at the solution treatment temperature (475 °C). The SEM-BSE images in Figure 7d–f and Figure 8d–f show that the η phases continue to dissolve into matrix. However, the SEM-BSE images of the SH samples in Figure 8e suggest that the η phases are almost dissolved before the holding process. It should be noted that, during heating, the EC values of the FH samples were always higher than the values of the SH samples. After solution treatment, similar EC values were found in both the FH and SH samples.

### 3.3. Recovery Prior to Recrystallization

**Figure 9:**
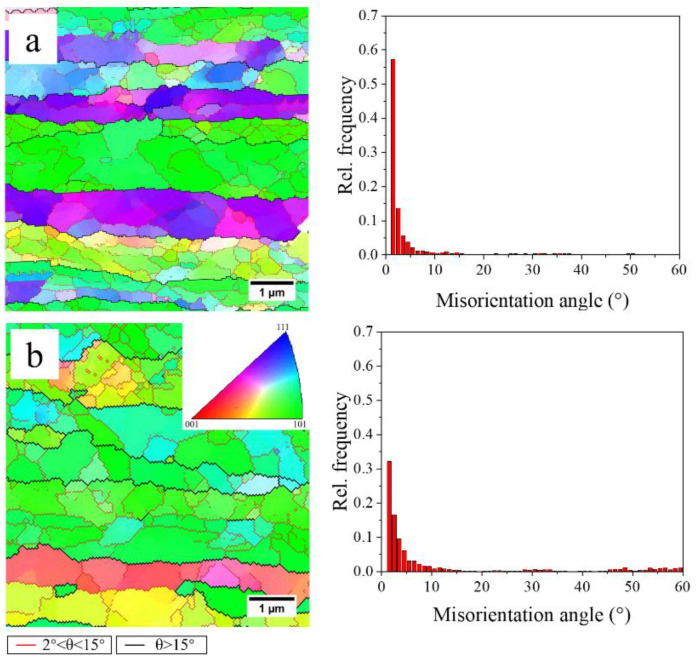
The EBSD maps and corresponding histograms of the misorientation angle distribution of samples quenched at 300 °C (**a**) FH, (**b**) SH.

When the samples are heated to the solution temperature, the recovery of the deformation structure takes place before the onset of recrystallization [19]. It is well known that most wrought aluminum alloys start to recrystallize at temperatures above 300 °C [19], but the exact temperature range depends on the wrought microstructure. Thus, the microstructures of samples quenched at 300 °C were characterized using EBSD to study the recovery prior to recrystallization during the fast and slow heating processes. The EBSD maps and corresponding histograms of the misorientation angle distribution are shown in Figure 9. The observed sub-grains in these samples indicate that recovery occurs during both fast and slow heating. The frequency of low-angle grain boundaries in the FH sample quenched at 300 °C is higher than that in the SH sample quenched at 300 °C. This suggests that the degree of recovery in the FH sample is less than that in the SH sample. The sub-grain sizes were also obtained by processing the EBSD data and the results show that the sub-grain sizes of the FH sample and the SH sample quenched at 300 °C are 137.0 ± 26.8 and 246.1 ± 51.0 nm, respectively. The coarser sub-grains confirm the strong recovery in the SH sample quenched at 300 °C.

The dislocation densities of samples quenched at 300 °C during the fast and slow heating process were also measured to study the degree of recovery or the driving force for the following recrystallization. The dislocation densities obtained from CMWP fitting are listed in Table 2. After rapidly heating (FH) to 300 °C (soaking 20 s at 475 °C), the dislocation density decreased from 3.28 ± 0.43 × 10^14^ m^−2^ in the cold-rolled sheet to 1.56 ± 0.31 × 10^14^ m^−2^. The dislocation density of the sample quenched at 300 °C during the slow heating process is 0.63 ± 0.16 × 10^14^ m^−2^. Compared to the rapid heating, a larger proportion of the stored energy of dislocations is consumed due to the strong recovery in the slow heating stage.

The heating time, dispersoids and precipitates (η phases) can all affect recovery during the heat-up stage of solution treatment. The heating time during the slow heating process is long enough for dislocations to rearrange, while the time for the fast heating is too short (20 s). The dispersoids and η phases can hinder the recovery by pinning the migration of dislocations and low-angle grain boundaries. The EC data in Figure 6 and SEM images in Figure 8 show that less η phases were left in the SH sample, exerting a weak pinning effect. The recovery during the slow heating is much stronger than that during fast heating. In other words, the driving force for the following recrystallization in the FH sample is higher.

### 3.4. Grain Structures and Textures after Solution Treatment

**Figure 10:**
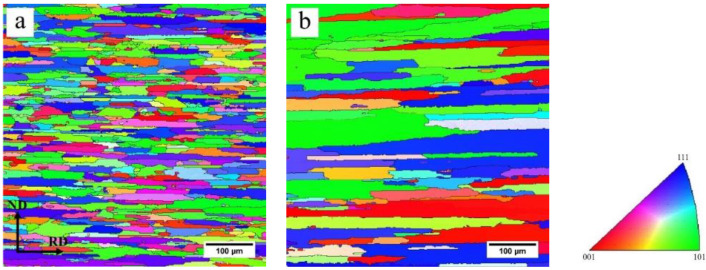
EBSD maps of samples after solution treatment; (**a**) FH, (**b**) SH, 50 °C/h.

Figure 10 shows the EBSD maps of the FH and SH samples after solution treatment. The EBSD results show the partial recrystallization in both the FH and SH samples. A rapid heating rate promotes a high degree of recrystallization (nearly 90%), while recovery significantly reduces the recrystallized grain fraction of a slowly heated sample (72.1%). The average grain size was obtained by processing the EBSD data with the Channel 5 software. The grain aspect ratios of samples were defined by D_RD_/D_ND_ to describe the elongated grain structure [29]. D_RD_ and D_ND_ measurements were performed along RD and ND on EBSD maps by a linear intercept method. The average grain sizes and aspect ratios are listed in Table 3. After solution treatment, the FH sample had a finer grain structure with the average grain size of 15.9 ± 3.7 μm. The grain size of the SH sample was approximately twice (28.8 ± 5.2 μm) that of the FH sample. Both the FH sample and SH sample presented elongated grain structures and the aspect ratios of the FH and SH samples were 4.4 and 6.0, respectively.

**Figure 11:**
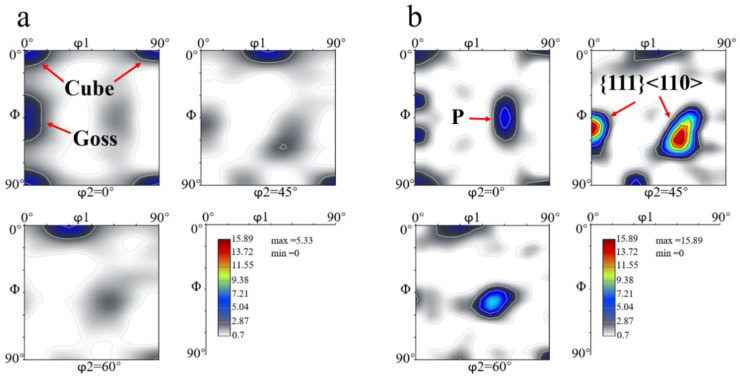
ODF maps (0°, 45° and 60°) of samples after solution treatment (**a**) FH, (**b**) SH.

The ODF maps of the FH and SH samples, measured using XRD, are shown in Figure 11. After solution treatment at 475 °C for 1 h, the texture components of both the FH sample and the SH sample consisted of the typical recrystallization textures, such as Cube, Goss, and P. The {111}<110> orientation, known as one of shear-type textures [30], was also found in the samples, but the sharpness of this texture in the FH sample was much lower than that in the SH sample. The intensity of the shear type {111}<110> texture in the SH sample was around 15.89 and it was the sharpest texture component in the SH sample. The Cube orientation with the intensity of 5.33 was the strongest texture in the FH sample. It is noted that the intensities of Cube and Goss in the FH and the SH samples were almost identical, but the P-type texture was stronger in the SH sample than in the FH sample. It is interesting that the Goss orientation in the SH sample, shown in Figure 11b, tends to split up.

### 3.5. Tensile Properties

**Figure 12:**
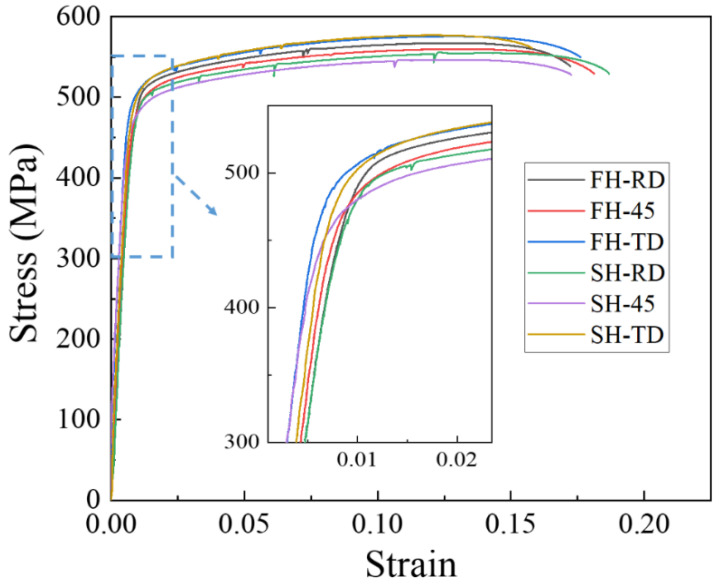
The tensile curves of the FH and SH samples after artificial aging (T6).

As the tensile curves of the three tests for every condition coincided with each other, the average strengths were calculated and are listed in Table 4. One of the stress–strain curves of the FH and SH samples, tested along RD, 45° from the RD and TD after artificial aging (T6), are shown in Figure 12. The inset figure in Figure 12 shows the elastic deformation stage with the yield point zoomed. The r-value and Δr were calculated according to the equation:

(1) $$r=\frac{\varepsilon_{w}}{\varepsilon_{t}},$$

(2) $$\Delta r=\frac{1}{2}\left|r_{\mathrm{RD}}{+r}_{\mathrm{TD}}{-2r}_{45}\right|$$

where ε_w_ and ε_t_ are the plastic strain in the sample width and thickness. r_RD_, r_TD_ and r_45_ represent the r-values obtained from the samples tested along the RD, transverse direction (TD) and 45° from the RD, respectively. The r-value was determined at a strain of 10%.

The main differences produced by the rapid heating and slow heating during the solution treatment are the grain sizes and textures. For Al-Zn-Mg-(Cu) alloys, however, grain refinement strengthening is not the dominant strengthening mechanism. Consequently, it is reasonable that there were no huge differences in yield strength and tensile strength between the FH and the SH samples after artificial aging to T6 temper. For both heating rates, the maximum yield strength and tensile strength occurred in the samples tested along TD. The variation of tensile strength between maximum (TD) and minimum (45° from the RD) strengths of the FH samples was around 17.1 MPa, while the value for the SH samples was ~29.0 MPa. A similar situation for the yield strength was also found. These results imply a higher anisotropy in the SH samples after artificial aging. The calculated r-values increased from the RD to 45° from the RD to the TD. The Δr for the FH samples was only 0.035 but the value for the SH samples was as high as 0.16. The anisotropic properties and the strong plastic anisotropy in the SH sample are attributed to strong textures after solution treatment [31].

## 4. Discussion

**Figure 13:**
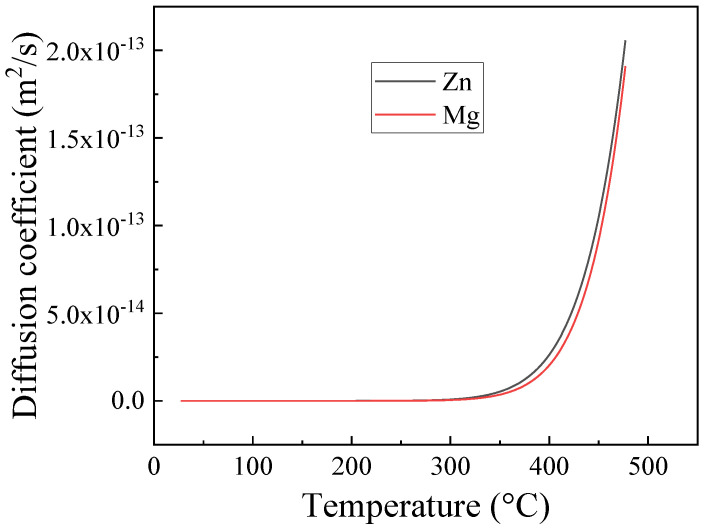
The diffusion coefficients of Zn and Mg change with temperature in the heating stage.

The dissolution of the η phase during the solution treatment is controlled by the diffusion temperature and time, which are related to the heating rates. The diffusion coefficients of Zn and Mg elements change with the temperature during the heating process, as shown in Figure 13.

It is clear that the diffusion coefficients of both Zn and Mg are small under 300 °C. It only took 20 s for the rapid heating to heat to 300 °C, so few η particles dissolved below 300 °C in the fast heating stage, resulting in the slight reduction in EC shown in Figure 6. However, the longer time (~5.5 h) of slow heating to 300 °C is sufficient, even at this lower temperature, to enable extensive precipitate dissolution, leading to a rapid decrease in EC, as shown in Figure 6. Although the heating time for the FH sample is still short, Figure 13 shows a rapid increase in the diffusion coefficients of both the Zn and Mg elements in the temperature range of 300–450 °C. The diffusion coefficients, or temperature, may be the dominant factor for the η phase to dissolve during heating from 300 to 450 °C. The EC values of both fast heating and slow heating, shown in Figure 6, decrease rapidly, and again, the long heating time leads to the lower EC value and less η phase of the SH sample. The EC values in Figure 6 and the SEM images in Figure 7a–d and Figure 8a–d suggest that the dissolution of the η phase mainly occurred at intermediate temperatures (300–450 °C) during the heating, especially at the slow heating rates. Any remaining precipitates, although few in number, were dissolved into the matrix in the following heat-up (450–475 °C) and 1 h soak at the solution treatment temperature (475 °C), since the diffusion is favored by both the temperature and time. This is confirmed by the slight decrease in EC in Figure 6 and the SEM images in Figure 7e,f and Figure 8e,f.

Recovery was found to occur during the heating processes, even at the fast heating rate. There is a competition between recovery and recrystallization in the consumption of stored energy. Recovery decreases dislocation density (stored energy) and reduces the number of viable nucleation sites for recrystallization. In our slowly heated samples, the dislocation densities in Table 2 and EBSD maps in Figure 9b indicate a strong recovery before recrystallization, decreasing the number of favorable nucleation sites and resulting in a larger grain size. For example, the gradient of dislocation density in the vicinity of the constituents is decreased during recovery, reducing the nucleation sites induced by the particle-stimulated nucleation (PSN). Similarly, other kinds of nucleation mechanisms are also influenced due to the consumption of stored energy by recovery. For the fast heating rate, the relatively high stored energy (less recovery) accelerates the nucleation, producing a large number of nucleation sites. This is the reason why the FH sample has finer grains after solution treatment. Bampton et al. [32] explored the effect of heating rate and precipitate dissolution on the recrystallization of the AA7075 sheet, but they did not assess the effects of recovery, although the effect of heating rate on the recrystallized grain size was similar to that observed in the present work. The higher EC of the FH samples during heating, shown in Figure 6, as discussed above, suggests that a higher volume fraction of η particles remained in the FH samples during recovery and recrystallization. Compared to the SH samples, therefore, a stronger interaction between the η particles and (sub)grain boundaries in the FH samples is another reason for the lesser recovery prior to recrystallization and the finer grains after solution treatment.

**Figure 14:**
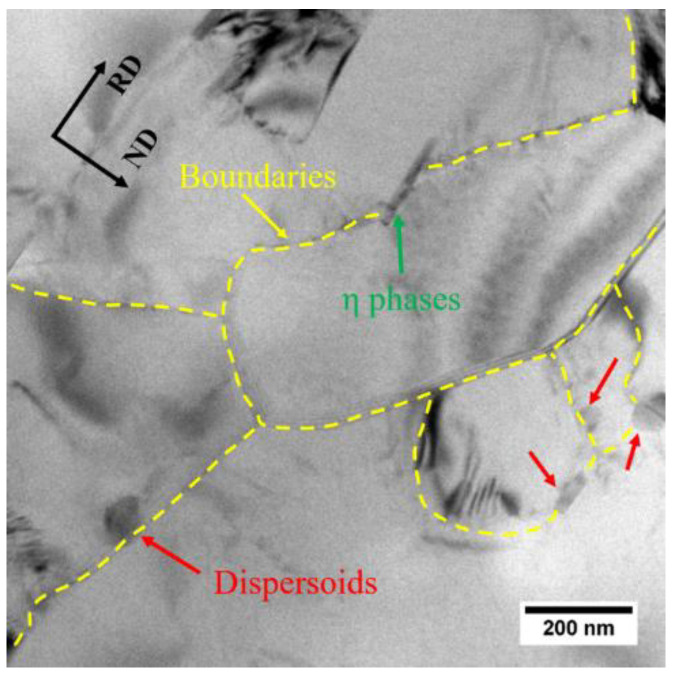
The TEM observation of the pinning effect of particles on boundaries in the FH sample quenched at 300 °C.

**Figure 15:**
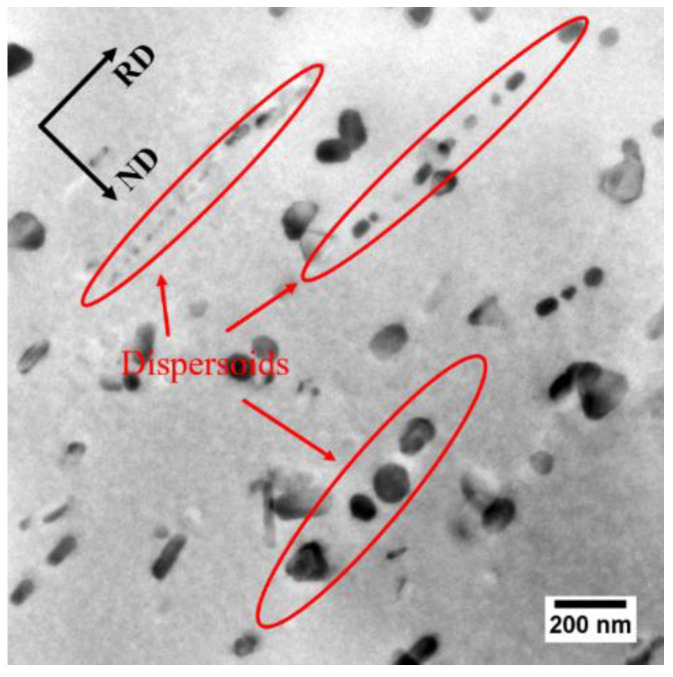
The distribution of dispersoids observed using TEM after solution treatment.

The elongated grains, shown in Figure 10, as well as the Cube, Goss, P and {111}<110> textures, shown in Figure 11, were observed in both the FH and SH samples after solution treatment. It has been reported that these kinds of structures are usually observed in aluminum alloys when the concurrent precipitation of dispersoids occurs during heat treatment [33,34]. As discussed above, however, the dissolution of η particles, rather than concurrent precipitation, occurs during the solution treatment, even at the slow heating rate. The elongated grains and textures can be attributed to the pinning effect of the dispersoids formed during homogenization and the undissolved η particles on the migration of the (sub)grain boundaries. In order to confirm this, the TEM observation was performed on the FH sample quenched at 300°C and the image is shown in Figure 14. The dispersoids and η particles are marked with red and green arrows, respectively. The η particles in Figure 14 have a needle-like shape with the longitudinal direction parallel to the RD. This shape results in a different pinning force on the migration of the (sub)grain boundaries along the RD and ND, helping to form the elongated grains. Dispersoids are insoluble and provide a constant pinning force to the (sub)grain boundaries during recovery and recrystallization. Figure 14 shows a smaller size of some of the dispersoids than that of the η particles, so the dispersoids are considered to be more effective in pinning the (sub)grain boundaries. The TEM observation of the solution treated sample, as shown in Figure 15, shows that the dispersoids are aligned in the RD. The difference of the pinning force on the migration of the (sub)grain boundaries, along the RD and ND is enhanced by the dispersoids. The migration of the (sub)grain boundaries along the ND is strongly blocked by the dispersoids and η particles, while the (sub)grains can easily grow along the RD. Thus, the elongated grains were produced after the solution treatment at both fast and slow heating rates.

The nucleation of recrystallization and grain growth are also competitive in consuming the stored energy. The nucleation of recrystallization is heterogeneous, indicating that the nucleation sites are not activated simultaneously at a certain temperature. During slow heating, the preferential nucleation sites were first activated at a relatively low temperature as the slow heating allows time for the grains to grow. The grain growth consumes the stored energy for the other kind of nucleation at higher temperatures in the following heating stage. In the case of rapid heating, the heating time is too short for grains to grow and the stored energy is retained for the nucleation in the following heating stage. For the slow heating, consequently, the variation of the pinning pressure on the migration of the (sub)grain boundaries along the RD and ND is amplified due to the long heating time or long moving distance of boundaries, resulting in the larger grain aspect ratio and grain size in the SH sample than in the FH sample. The higher stored energy of the dislocations for recrystallization nucleation is attributed to the finer grains in the FH sample.

## 5. Conclusions

- The heating rate influenced the dissolution of equilibrium η particles during the heating stage of the solution treatment and the final microstructure of the sheet at the time of recrystallization. The dissolution of η particles mainly happened during the heating process and these particles still remained when recrystallization began. Since the η particles gradually dissolved into the matrix, the recrystallization during heating process may be mainly affected by prior recovery and the sub-micron and indissoluble dispersoids.
- Recovery during the heating process consumes some of the stored energy for recrystallization. A high degree of recovery caused by the long time at low temperature results in the low nucleation rate for recrystallization and coarser grains in the slow-heated sample after solution treatment. The finer grains and higher recrystallized fractions in the fast-heated sample are attributed to more nucleation sites for recrystallization, due to the higher stored energy after recovery.
- The elongated nature of the recrystallized grains is caused by the variation in the pinning pressure of the migration of the boundaries toward the RD and ND, due to the shape and distribution of dispersoids and possibly η phases. The variation is enhanced by the long time for (sub)grain growth during the slow heating process, resulting in the strongly elongated grains.
- Typical recrystallization textures, such as Cube, Goss and P, as well as a shear {111}<110> texture, were found in both fast-heated and slow-heated samples after solution treatment. The sharp textures in the slow-heated sample led to the strong anisotropic tensile properties (high longitudinal yield strength, high Δr) after artificial aging to T6 temper.

## Figures and Tables

**Table 1:** Chemical composition of the AA7075 sheet.

Alloy	Zn	Mg	Cu	Cr	Mn	Ti	Fe	Si	Al
wt%	5.57	2.29	1.46	0.20	0.08	0.04	0.30	0.08	Bal.

**Table 2:** The dislocation densities of cold-rolled sample and samples quenched at 300 °C during fast and slow heating.

Status	Dislocation Density/10^14^ m^−2^	
	FH	SH
Cold-rolled	3.28 ± 0.43	3.28 ± 0.43
Quenched at 300°C	1.56 ± 0.31	0.63 ± 0.16

**Table 3:** The average grain size and grain aspect ratio of the FH sample and SH sample after solution treatment.

	Average Grain Size /μm	Grain Aspect Ratio
FH	15.9 ± 3.7	4.4
SH	28.8 ± 5.2	6.0

**Table 4:** Yield strength, tensile strength, and r-values along different tensile directions of the FH and SH samples after artificial aging (T6).

	Tensile Directions	Yield Strength/MPa	UTS/MPa	r	Δr
FH	RD	487.7 ± 4.9	567.4 ± 1.1	0.52	0.035
	45°	482.5 ± 4.9	559.9 ± 1.0	0.64	
	TD	508.9 ± 1.5	577.0 ± 6.4	0.69	
SH	RD	499.5 ± 3.7	554.8 ± 6.1	0.57	0.16
	45°	490.3 ± 5.2	546.5 ± 4.7	0.65	
	TD	511.9 ± 4.3	575.5 ± 2.3	1.05	
